# Moving Toward Patient-Centered Care in Africa: A Discrete Choice Experiment of Preferences for Delivery Care among 3,003 Tanzanian Women

**DOI:** 10.1371/journal.pone.0135621

**Published:** 2015-08-11

**Authors:** Elysia Larson, Daniel Vail, Godfrey M. Mbaruku, Angela Kimweri, Lynn P. Freedman, Margaret E. Kruk

**Affiliations:** 1 Department of Global Health and Population, Harvard T. H. Chan School of Public Health, Boston, MA, United States of America; 2 Epidemiology Department, Mailman School of Public Health, Columbia University, New York, NY, United States of America; 3 Ifakara Health Institute, Dar es Salaam, United Republic of Tanzania; 4 Averting Maternal Death and Disability Program, Mailman School of Public Health, Columbia University, New York, NY, United States of America; Örebro University, SWEDEN

## Abstract

**Objective:**

In order to develop patient-centered care we need to know what patients want and how changing socio-demographic factors shape their preferences.

**Methods:**

We fielded a structured questionnaire that included a discrete choice experiment to investigate women’s preferences for place of delivery care in four rural districts of Pwani Region, Tanzania. The discrete choice experiment consisted of six attributes: kind treatment by the health worker, health worker medical knowledge, modern equipment and medicines, facility privacy, facility cleanliness, and cost of visit. Each woman received eight choice questions. The influence of potential supply- and demand- side factors on patient preferences was evaluated using mixed logit models.

**Results:**

3,003 women participated in the discrete choice experiment (93% response rate) completing 23,947 choice tasks. The greatest predictor of health facility preference was kind treatment by doctor (β = 1.13, p<0.001), followed by having a doctor with excellent medical knowledge (β = 0.89 p<0.001) and modern medical equipment and drugs (β = 0.66 p<0.001). Preferences for all attributes except kindness and cost were changed with changes to education, primiparity, media exposure and distance to nearest hospital.

**Conclusions:**

Care quality, both technical and interpersonal, was more important than clinic inputs such as equipment and cleanliness. These results suggest that while basic clinic infrastructure is necessary, it is not sufficient for provision of high quality, patient-centered care. There is an urgent need to build an adequate, competent, and kind health workforce to raise facility delivery and promote patient-centered care.

## Introduction

The provision of high quality health care is central to improving population health and citizen satisfaction, and as such it is a key marker of successful health systems.[1] In its 2001 report, the Institute of Medicine defined high quality care as care that was safe, effective, patient-centered, timely, efficient, and equitable.[2] Patient-centered care is responsive to individual preferences, needs and values. It seeks to improve communication between the health worker and patient toward the aim of reducing information asymmetry and promoting care uptake and adherence. Making care more patient-centered can also lead to improvements in other areas of quality, particularly in safety and equity, leading to improved patient health status and satisfaction.[3]

The past two decades have witnessed sizeable changes in health in sub-Saharan Africa and other low-income regions: from the rapid rise of HIV/AIDS and subsequent partial success in controlling the epidemic, to the encouraging declines in child mortality and infectious diseases, to the growing burden of chronic disease.[4] Many of the poorest countries are beginning to experience the demographic transition, with declining fertility and rising life expectancy.[5] These shifts in health and demography are accompanied and sometimes fuelled by rapid urbanization and adoption of new lifestyles, including use of communication technology.[6] As health needs change and user expectations rise, health systems in low-income countries will need to adopt a patient-centered approach.[7,8]

In low-income countries struggling with high mortality from avertable causes, the focus has traditionally been on expanding provision of basic services, with less attention paid to interpersonal quality and responsiveness. Maternal mortality continues to be high in many countries: in Tanzania the maternal mortality ratio is 390 deaths per 100,000 live births.[9] Reducing maternal mortality requires that women deliver with a doctor, nurse, or midwife, ideally in a well-equipped healthcare facility.[10,11] Yet, according to the most recent Demographic and Health Survey (DHS) in Tanzania, although healthcare facility utilization increased from 2004 to 2010, it still remains low: in 2010 only 51.9% of women had delivered their most recent child in a healthcare facility. The problem does not appear to be a shortage of facilities as there is mounting evidence that women who do deliver in the health system are bypassing local clinics and delivering in facilities that they perceive as providing higher quality of care.[12–15] The growing evidence that women make active utilization choices in seeking care highlights the need to understand patient preferences for organizing effective maternal health services.[16]

Stated preference methods, such as discrete choice experiments, are one approach for understanding what users value in health care. Discrete choice experiments (DCEs) force users to make trade-offs among wanted attributes, thereby mimicking real life where not all preferences can be satisfied. The data from DCEs can then be used to quantify the relative importance of aspects of health care, providing useful information to policymakers with limited health budgets. In this paper we assess patient preferences for delivery care and examine how preferences vary for different healthcare users. We are particularly interested in assessing user characteristics that can be expected to change substantially in the future.

## Methods

### Study setting and participants

This discrete choice experiment was conducted as part of baseline data collection for a maternal and newborn health quality improvement project currently underway in four rural districts of Pwani Region, Tanzania (ISRCTN 17107760). The study areas are primarily rural and most residents are employed in small-scale subsistence farming or unskilled manual labor [17]. The 2010 DHS reported higher healthcare facility utilization than the national average, with 74.9% of women utilizing a healthcare facility for their most recent birth.[17]

The study includes 24 government-run primary healthcare facilities and their designated catchment villages.[12,18] For the population-based survey and discrete choice experiment (DCE) we conducted a full census of all households in the designated catchment areas. All women who were at least 15 years of age and delivered between six weeks and one year prior to the interview were invited to participate in a structured interview, including a DCE. If eligible women were not available on the day interviewers visited, at least two additional visits were made to reach the women. Participants were included in the current analysis if they completed at least part of the DCE.

All eligible women were informed of the purpose of the study and their right to refuse to participate. All interviewed participants provided written consent, or in the case of minors, their written assent and guardian written consent. The study was approved by the ethics review boards at Columbia University and Harvard University in the U.S., as well as the Ifakara Health Institute and the Tanzanian National Institute for Medical Research in the United Republic of Tanzania.

### Study instruments

#### DCE design

In order to identify potential delivery care attributes that were important to women we conducted a review of the literature and held meetings with policy-makers, healthcare workers, and health systems researchers in Tanzania. We then held five focus groups, each with six to eight women who had delivered a child in the past year and lived within the study districts, but not in catchment villages. Trained facilitators led the focus group discussions; participants provided informed consent. During these focus group discussions women were asked to identify important features of delivery care that they used to select a delivery facility and to distribute 20 token resources (stickers) among a list of care attributes. We focused on attributes that represented inputs and processes of care, as these elements can be directly addressed by implementers and are less likely to dominate other answer choices than an outcome attribute, such as life birth. We selected the six healthcare facility attributes that women ranked as most important when distributing their resources for inclusion in the DCE. We confirmed that women also discussed these attributes during the focus group discussions as characteristics they consider when choosing a delivery facility.

A local artist created graphics for each attribute level in order to facilitate understanding in this low-literacy population. The full survey and the discrete choice experiment were then piloted with 40 women living outside the study catchment area who had delivered a child in the six weeks to one year prior to pilot. We used information from the pilot survey (e.g. how much the women had paid for their most recent delivery) as well as information from the DCE and conversations with the women after the pilot to refine the DCE, improving clarity, and ensuring locally relevant range levels for the price paid for the delivery, hereafter referred to as “cost.” The six selected DCE attributes were: kind treatment by the health worker, healthcare facility privacy, healthcare facility cleanliness, modern equipment and medicines, health worker medical knowledge, and cost. The health workers were referred to as “doctor” in the DCE as this was the locally appropriate label. However, most healthcare providers in the primary care clinics in this region are clinical officers, nurses, or medical attendants.

All attributes were dichotomous except cost, which had five levels. This gives rise to 160 possible facility combinations in the full factorial design (5^1^X2^5^). In order to minimize the burden on respondents while maximizing efficiency, we selected five sets of eight choice scenarios (choice tasks). This was done using an experimental design that minimizes overlap of attribute levels within each task, maximizes level balance such that different attribute levels appear with approximately equal frequency across tasks, and achieves orthogonality among attributes by selecting levels for each attribute independently (Sawtooth Software Inc., Sequim, WA, USA). The average attribute efficiency was 0.95 (range: 0.86–0.98). This suggests a highly efficient design.

**Fig 1:**
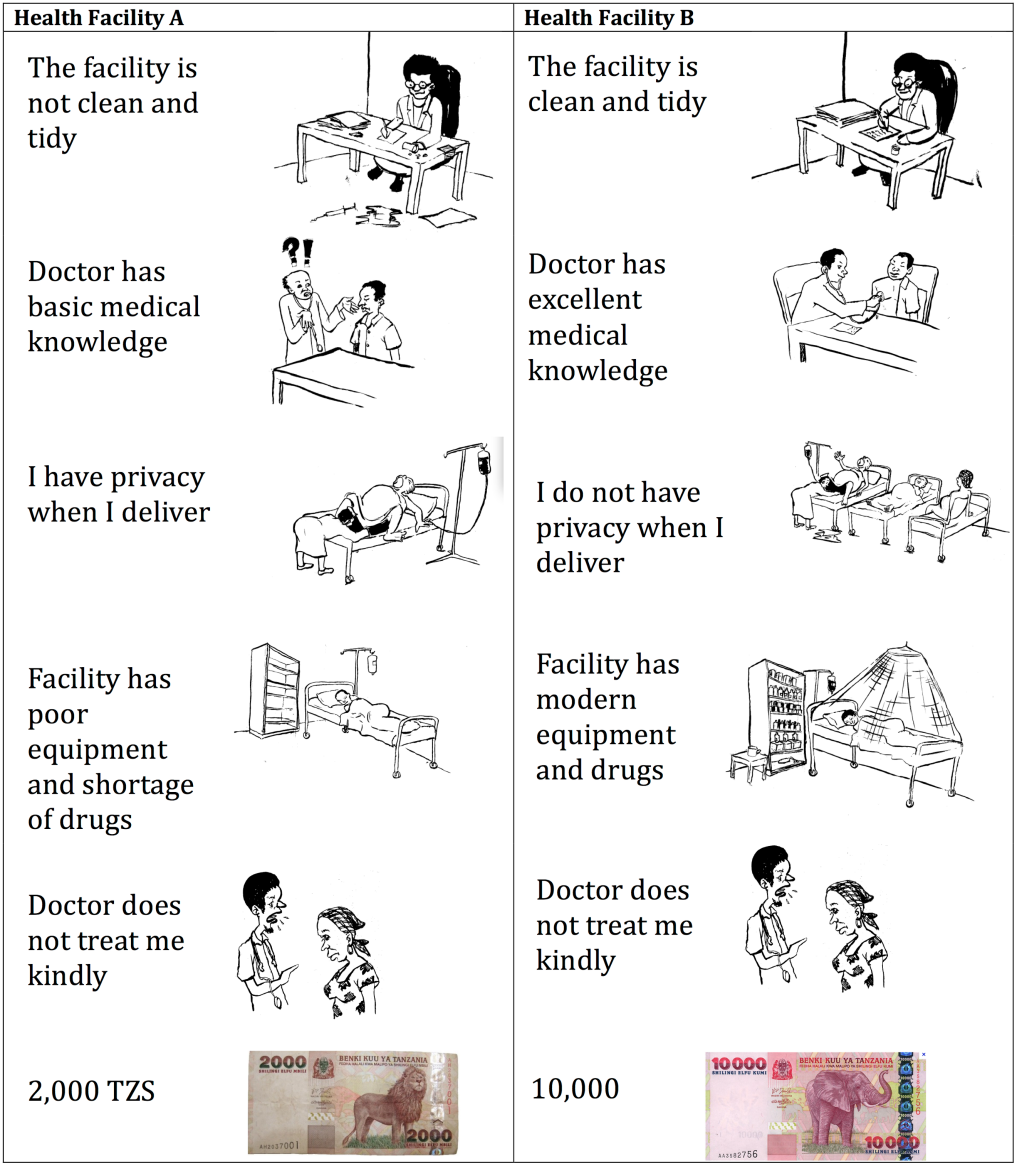
Sample DCE scenario card, 2012.

The final DCE included eight experimental choice tasks plus a fixed choice task that was kept constant across all five sets and used to test the internal predictive validity of the model. Each respondent was thus presented with nine choices. The final set of attributes and their levels are outlined in Table 1 and a sample scenario is shown in Fig 1.

**Table 1:** Attributes and levels for the discrete choice experiment.

Attribute	Levels
Medical equipment & drugs	Facility has modern equipment and drugs
	Facility has poor equipment and shortage of drugs
Doctor's medical knowledge	Doctor has excellent medical knowledge
	Doctor has basic medical knowledge
Doctor's attitude	Doctor treats me kindly
	Doctor does not treat me kindly
Facility cleanliness & organization	The facility is clean and tidy
	The facility is not clean and tidy
Privacy	I have privacy when I deliver
	I don't have privacy when I deliver
Cost, TZS	2,000 TZS; 5,000 TZS; 10,000 TZS; 20,000 TZS; 30,000 TZS

#### Non-DCE Variables

The population-based survey collected information on women’s demographics, household characteristics, maternal and newborn health experiences and health system use and satisfaction. We used these data to assess how individual factors and health system experiences influenced women’s stated preferences.

We assessed demographic and household characteristics including age, education (any secondary education versus less education as women with secondary education have been shown to be more selective users of health care), media exposure (index ranging from 1–9 constructed from frequency of exposure to newspapers, radio, and television), and primiparity (as primiparous women demonstrate different utilization patterns [19,20]). Using principal components analysis of a set of 18 questions on ownership of household assets we constructed a relative wealth index [21]. We compared the wealthiest 20% of women to all others as wealthier women may have increased access to facilities, which may shape their expectations and preferences.

Because a woman’s experiences may affect her expectations for care and her preferences, we assessed her recent experiences, including place of most recent delivery (healthcare facility versus home), number of antenatal care (ANC) services received for most recent delivery (a standardized index of 8 items: weight, height, blood pressure, urine sample, blood sample, malaria prophylaxis, tetanus vaccine, and iron supplements), and number of services received during most recent delivery (a standardized index of nine recommended services: mother checked; baby checked; uterotonic received; and mother given advice on: immediate feeding, exclusive breastfeeding, umbilical cord care, washing hands, immunization, and how to avoid chilling the baby).

Additional supply-side factors included distance from woman’s hamlet to the nearest hospital (spherical distance from the center of the hamlet to the hospital using the sphdist command in Stata 13.1) and the density of secondary health facilities within 25km of the woman’s hamlet. For distance calculations when the hamlet location was unknown the village center was substituted (23.5%).

### Data collection

The survey was conducted face-to-face in Swahili by six teams of five local interviewers. All interviewers underwent 11 days of training, including one full day of DCE training and practice.

Each choice task was presented to the respondent using a single sheet of paper (Fig 1). The interviewer read a standardized script that asked the respondent to imagine that she was pregnant and given the choice between delivering at the two facilities presented. She was asked to choose at which healthcare facility she would prefer to deliver, and was reminded that there was no right or wrong choice and that her answers would be confidential. Her responses were recorded using hand-held tablet computers. Data collection occurred between February 13 and April 28, 2012.

### Statistical analysis

Data were imported into Stata version 13.1 (StataCorp LP, College Station, USA) and were examined for outliers and missingness. Descriptive statistics were calculated for the non-DCE variables of interest. The attribute utilities were estimated with a mixed logit model using 500 Halton draws, with normally distributed parameters and an independent covariance structure. Conditional logit models allow respondents’ unselected alternative choices in each task to be taken into account when estimating preferences, as well as individual-specific characteristics. The mixed logit model is an extension of the standard conditional logit model that allows for attribute coefficients to be randomly distributed [22]. Mixed logit models are frequently used to analyze DCEs [23–25].

To assess the potential effects of both supply- and demand- side characteristics on the utility of each attribute, we analyzed a set of models that introduced individual characteristics as an interaction between that characteristic and the attributes of the facility. Cost was specified as a fixed effect in each model and all other clinic attributes were random [26]. We were particularly interested in characteristics that can be expected to change in the future. We assessed interactions with the demographic characteristics of wealth, secondary education, and primiparity. We also assessed an interaction with the women’s exposure to media, as this may influence her preference for delivery. Finally, because a woman’s experiences may affect her expectations for care and her preferences, and because facility utilization for delivery is increasing in Tanzania, we assessed an interaction with the place of most recent delivery (healthcare facility versus home).[17,27]

We performed several validity tests, including evaluating the in-experiment predictive validity of the mixed logit model. The internal predictive validity of our model was assessed by comparing women’s predicted vs. actual choices on the fixed choice card (which is excluded from the sample used to estimate the mixed logit model). The fixed choice card offered women the option of selecting a healthcare facility with desirable nontechnical attributes (a kind doctor, privacy, and a clean and tidy facility) or a facility with desirable technical attributes (modern equipment and drugs, and a doctor with excellent medical knowledge). We report the actual choice and mean predictive values.[28]

All analyses were conducted using Stata 13.1 (StataCorp LP, College Station, USA).

## Results

Of the 3,238 eligible women, 3,019 (93%) consented and were interviewed. 3,003 women participated in the DCE; 2,953 women completed all eight DCE choice cards included in the mixed logit regression and 2,950 women completed all eight choice cards plus the fixed choice card.

The average age of respondents was 27.1 years old. 82.7% were currently married or living with their partner, 60.7% listen to the radio daily, and 24.0% were primiparous (Table 2). Women lived on average 38.7 km from the nearest hospital. Most women (72.0%) delivered their most recent child in a health facility.

**Table 2:** Demographic characteristics of women delivering a child in the 6 weeks to 12 months prior to interview, Pwani Region, Tanzania, 2012 (n = 3,003).

**Maternal factors**	**n**	**(%)**
Age, mean (SD)	27.1	(6.8)
Muslim^a^	2434	(81.1)
Currently married^b^	2483	(82.7)
Education		
No formal education	799	(26.7)
Any primary	1919	(64.0)
Any secondary	280	(9.3)
Primary occupation, farmer or homemaker^c^	2426	(82.0)
Listens to the radio daily	1824	(60.7)
Owns own mobile phone	1071	(35.8)
Household has electricity	188	(6.3)
Self-rated health, good^d^	2068	(68.9)
Primiparous	722	(24.0)
**Maternal health experiences**		
Has health insurance	164	(5.6)
> 5 visits to local clinic in past year	829	(29.4)
> 3 antenatal care visits for most recent pregnancy	1884	(63.2)
No. of antenatal services received for most recent pregnancy (max 9), mean(SD)^e^	7.0	(1.6)
Facility delivery for most recent birth	2133	(72.0)
No. of delivery services received for most recent delivery (max 9), mean(SD)^f^	6.5	(2.1)
Experienced disrespect and abuse during most recent delivery	166	(7.8)
Perceived overall quality of care at local clinic as very good or excellent versus good, fair, or poor	965	(34.0)

^a^ 17.9% Christian

^b^ Includes legal marriages and cohabitating couples

^c^ Includes homemakers, farmers, and house cleaners

^d^ Based on a "no problem" rating for all five items (mobility, self-care, usual activities, pain or discomfort, and anxiety or depression) of the EQ-5D instrument EuroQol Group, Rotterdam, Netherlands)

^e^ Index of nine antenatal care services: diagnostics, treatment, and counseling

^f^ Index of nine delivery services: diagnostics, treatment, and postnatal counseling.

For the base model (without interactions) 23,947 choice tasks were analyzed. Table 3 summarizes the findings from the DCE for both the base model and models with interaction terms. In the base model, the variable with the greatest magnitude of association with healthcare facility preference was respectful treatment by the doctor (β = 1.13, p<0.001), followed by a doctor with excellent medical knowledge (β = 0.89, p<0.001) and modern medical equipment and drugs (β = 0.66, p<0.001). As expected, respondents demonstrated a negative preference for cost (β = -0.01, p<0.001).

**Table 3:** Mixed logit model results with and without interactions for a discrete choice experiment addressing healthcare facility preferences for delivery among women in Pwani Region, Tanzania, 2012.

	Base model		w/ primiparity interaction		w/ some secondary education interaction		w/ wealthiest quintile interaction		w/ media exposure interaction		w/ facility utilization (any facility v. home) interaction	
Attribute	β^a^	p-value	β^a^	p-value	β^a^	p-value	β^a^	p-value	β^a^	p-value	β^a^	p-value
Facility has modern medical equipment & drugs	0.66*	<0.001	0.65*	<0.001	0.65*	<0.001	0.64*	<0.001	0.53*	<0.001	0.62*	<0.001
Doctor has excellent medical knowledge	0.89*	<0.001	0.89*	<0.001	0.87*	<0.001	0.87*	<0.001	0.62*	<0.001	0.84*	<0.001
Doctor treats patient kindly	1.13*	<0.001	1.13*	<0.001	1.15*	<0.001	1.10*	<0.001	1.05*	<0.001	1.18*	<0.001
Facility is clean and tidy	0.34*	<0.001	0.31*	<0.001	0.33*	<0.001	0.33*	<0.001	0.31*	<0.001	0.26*	<0.001
Patient has privacy for delivery	0.29*	<0.001	0.24*	<0.001	0.27*	<0.001	0.27*	<0.001	0.21*	<0.001	0.15*	0.001
Cost, TZS^b^	-0.01*	<0.001	-0.01*	<0.001	-0.01*	<0.001	-0.01*	<0.001	-0.02*	<0.001	-0.01*	<0.001
**Interaction terms**												
Facility has modern medical equipment & drugs X covariate	-	-	0.01	0.816	0.08	0.374	0.07	0.280	0.04*	0.005	0.06	0.319
Doctor has excellent medical knowledge X covariate	-	-	0.03	0.658	0.28*	0.003	0.16*	0.024	0.09*	<0.001	0.09	0.153
Doctor treats patient kindly X covariate	-	-	0.01	0.947	-0.18	0.134	0.18*	0.043	0.03	0.150	-0.07	0.379
Facility is clean and tidy X covariate	-	-	0.13*	0.019	0.11	0.166	0.04	0.515	0.01	0.490	0.11*	0.030
Facility has privacy X covariate	-	-	0.21*	<0.001	0.21*	0.016	0.10	0.092	0.03	0.053	0.20*	<0.001
Cost X covariate	-	-	0.00	0.176	0.01	0.096	0.00	0.566	0.00	0.069	-0.00	0.845
No. of respondents	3,003		3,003		3,002		2,992		3,001		2,964	
No. of choice tasks	23,947		23,947		23,939		23,859		23,931		23,639	
Log-likelihood	-13,357		-13,345		-13,339		-13,301		-13,324		-13,159	
Likelihood ratio **χ** ^2^	1,225		1,224		1,212		1,212		1,197		1,214	

^b^ Cost in 1000 TZS

^a^ The mean relative utility of each attribute conditional on the additional attributes in the choice set. Standard deviations are not shown for ease of readability.

*Significant at α = 0.05.

The results from four interaction models are presented in Table 3. Additional interaction models can be found in the S1 Table. Primiparous women showed a higher preference for privacy and cleanliness of facility than women who had delivered more children, as did women who delivered in a health facility compared to women who delivered at home. Privacy was also more important to women who had some secondary education compared to women without secondary education. Women with some secondary education showed more preference for doctors with excellent medical knowledge compared to women with less education. This was also true for women with increased media exposure and wealthier women. Preference for modern medical equipment and drugs increased with increased media exposure.

In assessing the predicted versus actual choice of facility in the fixed choice card, the predicted 53.2% of women would select the facility with desirable nontechnical attributes. In the actual experiment, 55.9% of women selected this facility.

## Discussion

While availability of modern medical equipment and drugs, facility cleanliness, and privacy were all significantly associated with choosing a health facility for obstetric care, this group of rural Tanzanian women valued health worker kindness above other attributes of the health facility, followed closely by excellent medical knowledge. Kind treatment by a health worker, as well as her knowledge and skills, is demonstrated through the process of delivery care; that is, the information is gleaned by women from the interaction with the health care worker. These relational aspects of quality were more important than inputs, such as equipment or cleanliness. This is supported by previous research with this cohort of women that found that ratings of quality of care relied heavily on the process of care and nature of the services received, rather than infrastructure.[18]

The joint emphasis on technical and non-technical quality of care is consistent with findings from a prior discrete choice experiment conducted among women in Kigoma Region, Tanzania in 2007, which also found that an attentive health worker and equipment availability were the most important features of the health facility when making the decision for place of birth.[20] The similarity of preferences is noteworthy given the different context in Kigoma, where facility delivery was substantially lower (37% vs. 72% here) and respondents were less educated. A DCE with similar attributes to ours asked adults in Zambia asked to select a hospital for cerebral malaria care. Their study found that ‘appropriate exam’ was the most important attribute of hospital care while followed by ‘provider kindness’, further supporting this notion that the quality of the process of care is important to patients.[29]

In order to understand how delivery care preferences may be affected by changing economic, social, and demographic shifts we analyzed interactions between several maternal characteristics and the facility attributes in the DCE. Penetration of mobile phones and access to media are increasing and the country and region are growing economically.[30,31]

We found that more educated women cared more about the medical knowledge of the health worker and privacy than women without any secondary school education. The influence of education on preferences for facility characteristics is consistent with other papers from both low- and high-income countries that demonstrate links between education and satisfaction with healthcare.[32,33] Recent cross-country analyses have found that an individual’s level of education affects her rating of health system responsiveness on standardized vignettes.[34,35] This may be reflective of varied preferences and education. The proportion of women with secondary school education is increasing in Tanzania, and with increased education it is likely that these expectations will grow in importance. Woman with greater exposure to media had a higher preference for health worker knowledge and availability of modern equipment and drugs, potentially indicating the influence of urban mores and greater appreciation of the benefits of technology.

Women in the highest wealth quintile valued the healthcare provider’s medical knowledge and attitude even more than other women. Women with higher economic status are likely to have higher expectations for care. As economic standards rise in this population, health worker attitudes and competence are likely to become even more important to patients.

We found that primiparous women and women with past healthcare facility delivery had stronger preference for cleanliness and privacy than other women. Utilization of healthcare facilities for childbirth increased in Pwani Region from 59% in 2004 to 73% in 2010.[17,27] As facility delivery rates rise, it is likely that women will become more selective when choosing between facilities,[12] and new mothers may look for clean and private facilities.

In addition to being valued above all other attributes of the health facility, preference for health worker kindness was least likely to vary based on women’s characteristics. Kind and respectful treatment of women in healthcare settings is not only a fundamental right, but also signals high quality care.[36] Previous assessment in this and similar Tanzanian populations found that women who reported disrespect and abuse were less likely to report the care received as high quality and less like to report an intention to select the same facility for future deliveries.[18,37]

This study had several limitations. The study is only able to demonstrate women’s preference for the attributes and levels presented and assumes no major variables were omitted. [38] To guard against this source of bias we undertook detailed formative research, but it is possible that some meaningful variables were not included. We found that the mixed logit model functioned well for predicting choice of facility in the fixed choice task—a test of experimental predictive validity. However, the findings here reflect preferences for a hypothetical facility choice. Because of the limited number of attributes in the study and the constrained facility choices in rural Tanzania, the facilities in the experiment do not precisely reflect the current situation of the health system in Tanzania and women’s preferences here may thus not be accurate in predicting actual utilization. In addition, this study only looked at women’s preferences for care. If husbands or other family members strongly influence choice, women’s preferences may not fully predict actual utilization. This study did not distinguish between a woman’s initial choice in health facility and her preference for referral facilities. It is possible that the likelihood of her complying with a referral would also be affected by the attributes of the referral facility. Finally, these results may not be generalizable to populations outside of this region. However, it is encouraging that the direction of these preferences was not different from other similar studies.[20,29]

This study of over 3,000 women points to the ingredients for patient-centered maternal health services. Investment by policymakers and implementers in competent and kind providers will improve the health system’s responsiveness to women’s needs and expectations. The emphasis put on high quality technical and non-technical care will only grow as communities in low-income countries become wealthier, better educated, and more exposed to urban norms. There is thus an urgent need to explore short- and long-term approaches for improving quality in resource-constrained health systems. Although health needs and resources differ, building a patient-centered health system is a shared health system challenge for rich and poor countries.

## Supporting Information

S1 Table Additional mixed logit model results with and without interactions for a discrete choice experiment addressing healthcare facility preferences for delivery among women with recent deliveries, Pwani Region, Tanzania. (DOCX)

S1 Dataset Data file used in analysis (Stata format version 13). (XLS)
