# Supplementary material for: Moving Toward Patient-Centered Care in Africa: A Discrete Choice Experiment of Preferences for Delivery Care among 3,003 Tanzanian Women
Source: PLoS One. 2015 Aug 11;10(8):e0135621. doi: 10.1371/journal.pone.0135621 (PMC4532509; doi:10.1371/journal.pone.0135621)
Supplement: S1 Table — (DOCX) [file pone.0135621.s001.docx]

**S1 Table.** Additional mixed logit model results with and without interactions for a discrete choice experiment addressing facility preferences for delivery among women with recent deliveries, Pwani Region, Tanzania, 2012

|  | **Base model** | | **w/ distance to nearest hospital interaction** | | | | **w/ delivery services received interaction** | | | | | **w/ ANC services received interaction** | | | | | |  |  |  |  |
| --- | --- | --- | --- | --- | --- | --- | --- | --- | --- | --- | --- | --- | --- | --- | --- | --- | --- | --- | --- | --- | --- |
| **Attribute** | **β^a^** | **p-value** | **β^a^** | | **p-value** | | **β^a^** | | | **p-value** | | **β^a^** | | | **p-value** | | |  |  |  |  |
| Facility has modern medical equipment & drugs | 0.66* | <0.001 | 0.80* | | <0.001 | | 0.45* | | | <0.001 | | 0.70* | | | <0.001 | | |  |  |  |  |
| Doctor has excellent medical knowledge | 0.89* | <0.001 | 0.91* | | <0.001 | | 0.57* | | | <0.001 | | 0.67* | | | <0.001 | | |  |  |  |  |
| Doctor treats patient kindly | 1.13* | <0.001 | 1.11* | | <0.001 | | 1.28* | | | <0.001 | | 1.42* | | | <0.001 | | |  |  |  |  |
| Facility is clean and tidy | 0.34* | <0.001 | 0.28* | | <0.001 | | 0.27* | | | 0.002 | | 0.40* | | | <0.001 | | |  |  |  |  |
| Patient has privacy for delivery | 0.29* | <0.001 | 0.40* | | <0.001 | | 0.42* | | | <0.001 | | 0.44* | | | <0.001 | | |  |  |  |  |
| Cost, TZS^b^ | -0.01* | <0.001 | -0.01* | | <0.001 | | -0.02* | | | 0.001 | | -0.02* | | | 0.009 | | |  |  |  |  |
| **Interaction terms** |  |  |  | |  | |  | | |  | |  | | |  | | |  |  |  |  |
| Facility has modern medical equipment & drugs X covariate | - | - | -0.004* | | 0.009 | | 0.03* | | | 0.018 | | -0.01 | | | 0.731 | | |  |  |  |  |
| Doctor has excellent medical knowledge X covariate | - | - | 0.00 | | 0.921 | | 0.06* | | | <0.001 | | 0.04* | | | 0.040 | | |  |  |  |  |
| Doctor treats patient kindly X covariate | - | - | 0.00 | | 0.800 | | -0.03 | | | 0.187 | | -0.04 | | | 0.083 | | |  |  |  |  |
| Facility is clean and tidy X covariate | - | - | 0.00 | | 0.182 | | 0.02 | | | 0.220 | | -0.01 | | | 0.558 | | |  |  |  |  |
| Facility has privacy X covariate | - | - | -0.003* | | 0.032 | | -0.01 | | | 0.407 | | -0.02 | | | 0.174 | | |  |  |  |  |
| Cost X covariate | - | - | 0.000 | | 0.573 | | 0.00 | | | 0.520 | | 0.00 | | | 0.740 | | |  |  |  |  |
| No. of respondents | 3,003 |  | 2,924 | |  | | 2081 | | |  | | 2815 | | |  | | |  |  |  |  |
| No. of observations | 23,947 |  | 23,315 | |  | | 16,594 | | |  | | 22,454 | | |  | | |  |  |  |  |
| Log-likelihood | -13,357 |  | -12,984 | |  | | -9,166 | | |  | | -12,448 | | |  | | |  |  |  |  |
| Likelihood ratio **χ**^2^ | 1,225 |  | 1,191 | |  | | 803 | | |  | | 1,164 | | |  | | |  |  |  |  |
| a The mean relative utility of each attribute conditional on the additional attributes in the choice set | | | | | | | | | | | | | | | |  | | |  |  |  |
| b Cost in 1000 TZS  *Significant at α =0.05 | |  |  |  | |  | |  |  | |  | |  |  | | |  | |  |  |  |
